# Variation of Triterpenic Acids in 12 Wild *Syzygium formosum* and Anti-Inflammation Activity on Human Keratinocyte HaCaT

**DOI:** 10.3390/plants10112428

**Published:** 2021-11-10

**Authors:** Hyun-ah Park, Mi Yoon Kim, Nan-Young Lee, Jaeyoon Lim, Kyu-been Park, Chang-Kyu Lee, Van Dao Nguyen, Jaehan Kim, Jong-Tae Park, Jong-Il Park

**Affiliations:** 1 CARBOEXPERT Inc., Daejeon 34134, Korea; hapark@carboexpert.com (H.-a.P.); nylee@carboexpert.com (N.-Y.L.); kbpark@carboexpert.com (K.-b.P.); cklee@carboexpert.com (C.-K.L.); 2 Department of Biochemistry, College of Medicine, Chungnam National University, Daejeon 35015, Korea; miyoonkim@cnu.ac.kr; 3 Translational Immunology Institute, Chungnam National University, Daejeon 35015, Korea; 4 Department of Food Science and Nutrition, Chungnam National University, Daejeon 34134, Korea; daebakch9086@gmail.com (J.L.); jaykim@cnu.ac.kr (J.K.); 5 Biotechnology Faculty, Binh Duong University, Thu Dau Mot 820000, Vietnam; nvdaohou@gmail.com; 6 Department of Food Science and Technology, Chungnam National University, Daejeon 34134, Korea

**Keywords:** anti-inflammation, secondary metabolite, skin cell, *Syzygium formosum*, triterpenic acid

## Abstract

*Syzygium formosum* (Wall.) Masam leaf is known as a Vietnamese traditional herbal medicine used to treat atopic dermatitis and stomach ulcers. Recently, its potent anti-allergic effects were reported with possible active compounds analysis. Here, we collected *S. formosum* leaves from 12 wild trees and compared compositions of triterpenic acids (TA) with *Centella asiatica*. Anti-inflammatory activities of *S. formosum* leaf extract (SFLE) was compared with *C. asiatica* extract (CAE) using human keratinocyte, HaCaT. In this study, up to seven TAs were identified in SFLE, while only madecassic and asiatic acids were detected in the CAE. Total TA content varied among SFLE, but asiatic, corosolic, and betulinic acids were the major components. Surprisingly, wild tree sample 12 (S12) contained total TA of 27.2 mg/g dry-leaves that was 5-fold greater than that in the *C. asiatica* sample, and S4 had the highest content of asiatic acid (12.6 mg/g dry-leaves) that accounted for 50% of the total TA. S4 and S12 showed more than 3-fold higher anti-oxidative power than the CAE. In the UVB irradiation model, S4 and S12 (5 μg/mL) strongly repressed mRNA levels of pro-inflammatory cytokines (IL-1β, IL-6, and IL-8) and COX-2, while the CAE at the same condition showed moderate or weak repression. The difference in anti-inflammation effects between the SFLE and the CAE was also confirmed by protein quantifications. Taken together, SFLE has great potentials as a new cosmeceutical ingredient with a higher amount of skin-active phytochemicals.

## 1. Introduction

Plant extracts are common source for the bioactive compounds that have been used in traditional remedies for human diseases. Medicinal herbs are developed in different countries and consumed as teas or can be applied on the disordered skin area [1,2]. Among them, Korean ginseng is one of the most popular plant used by ingestion, while *Centella asiatica* extract is the most successful active ingredient for cosmetics. Aloe vera and *Camellia* genus are also well-known functional plant for topical uses. Those plants have been intensively investigated for the identification of active phytochemicals and evaluations of biological activities.

*C. asiatica* extract contains various triterpene derivatives such as asiatic acid, madeccassic acid, asiaticosides, and madecasosides [3,4]. Those triterpene derivatives are regarded as hallmarks of functional phytochemicals effective on the various diseases or skin conditioning. Anti-inflammation, anti-atopic dermatitis, anti-melanogenesis, and collagen regeneration effects have been demonstrated in previous research [5,6,7,8]. Now, *C. asiatica* has become the most popular herb used as a cosmeceutical ingredient.

*Syzygium formosum* (Wall.) Masam leaf gained attention recently for its strong anti-allergic function in a mouse food allergy model [9]. The plant generally grows in subtropical area of South Asia. In Vietnam, *S. formosum* has been used as a traditional remedy to treat atopic dermatitis or gastritis. Its leaves are used as a tea: leaves of *S. formosum* are dried, then boiled with water. A relatively large amount of *S. formosum* tea is recommended to treat allergic or inflammatory diseases [9]. A recent study revealed that *S. formosum* leaf contains a large amount of triterpenic acids such as asiatic acid, corosolic acid, and betulinic acid [10]. There are many other phytochemicals, but those triterpenic acids that are already well investigated should be the major functional phytochemicals. Previous research had scientifically proven the value of *S. formosum* as a functional ingredient for food and cosmetic industries. However, incomplete information on the variations of phytochemicals by individual trees can hinder industrial applications of the tree. Moreover, standardized cell-basis assays are essential to evaluate *S. formosum* as a cosmeceutical ingredient.

Skin aging is a natural process but could be greatly affected by exposure of skin to sunlight. Ultraviolet (UV) is one of the most harmful types of radiation and induces dramatic photoaging that is accompanied by wrinkles, strong pigmentation, and even skin cancers. UVB (280–320 nm of wave length) is 5–10% of sunlight UV, but UVB exposure may be the reason of serious skin damages by oxidation of lipids and proteins and further inflammatory cascades [2,11]. A human dermal keratinocyte, HaCaT, has been used to evaluate UVB effects on human skin cell, even though the cell line has dysfunctional p53 and shows subtle differences when compared with primary human dermal keratinocyte to the UV radiation [12,13].

In this study, 12 wild trees of *S. formosum* were collected to investigate variations of triterpenic acids (TA). The leaves were dried and extracted with 70% ethanol. TA compositions were compared with that in *C. asiatica*, and functionalities of *S. formosum* leaf extract (SFLE) were evaluated by using a human keratinocyte, HaCaT cells. To induce inflammatory stress on the cells, UVB irradiation was used. Our study demonstrated potentials of the *S. formosum* leaf as a distinctive cosmeceutical ingredient with strong biological activities on skin.

## 2. Results

### 2.1. Triterpenic Acid Compositions of 12 Wild S. formosum Trees

**Figure 1:**
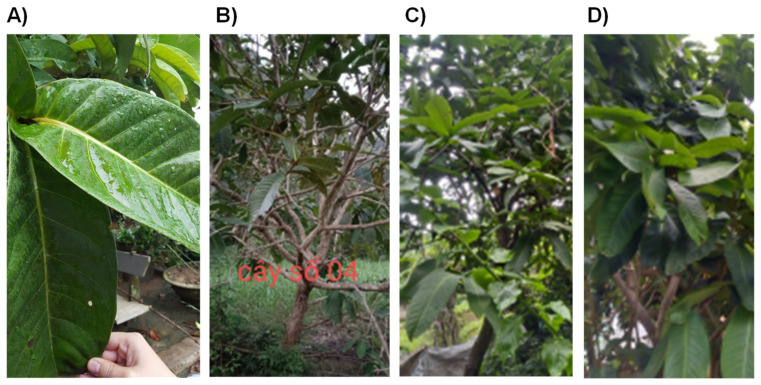
Examples of *S. formosum* leaf and trees. The size of the matured leaf could be estimated by comparison with a hand (**A**). Photos were taken just before harvesting leaves of each tree: (**B**) of S4, (**C**) of S10, and (**D**) of S12.

More than 30 wild trees of *S. formosum* were found in Hanoi and the suburbs of Hanoi, Vietnam, which is a typical subtropical area in South-East Asia. The young trees had relatively small leaves which were not proper to harvest, therefore, we selected trees older than 5 years and harvested at early morning to noon. The trees had very large leaves, up to 40 cm long (Figure 1). Old trees had thicker and bigger leaves than young trees. The color of the leaves does not change by seasons in the growth area.

#### 2.1.1. Variations of Triterpenic Acids

The twelve wild trees of *S. formosum* contained a significant amount of TAs in their leaves. Seven TAs including madecassic acid, asiatic acid, maslinic acid, colosolic acid, betulinic acid, oleanolic acid, and ursolic acid are commonly detected in the 12 trees (Table 1). Asiatic acid was the most present of the TAs, representing nearly a half of the total TA composition in some trees such as S4 and S11. The total amount of TAs varied by trees significantly, and showed more than 3-fold differences when minimal (S7) and maximal (S12) were compared. Either betulinic acid or colosolic acid was the second most represented TA in 12 trees of *S. formosum*. Others were relatively minor in the TA composition. Intriguingly, the total amount of TAs in *S. formosum* leaves was much greater than that of the *C. asiatica*. S12 had at least 5-fold greater amount of the total TAs than the *C. asiatica* (Table 1).

**Figure 2:**
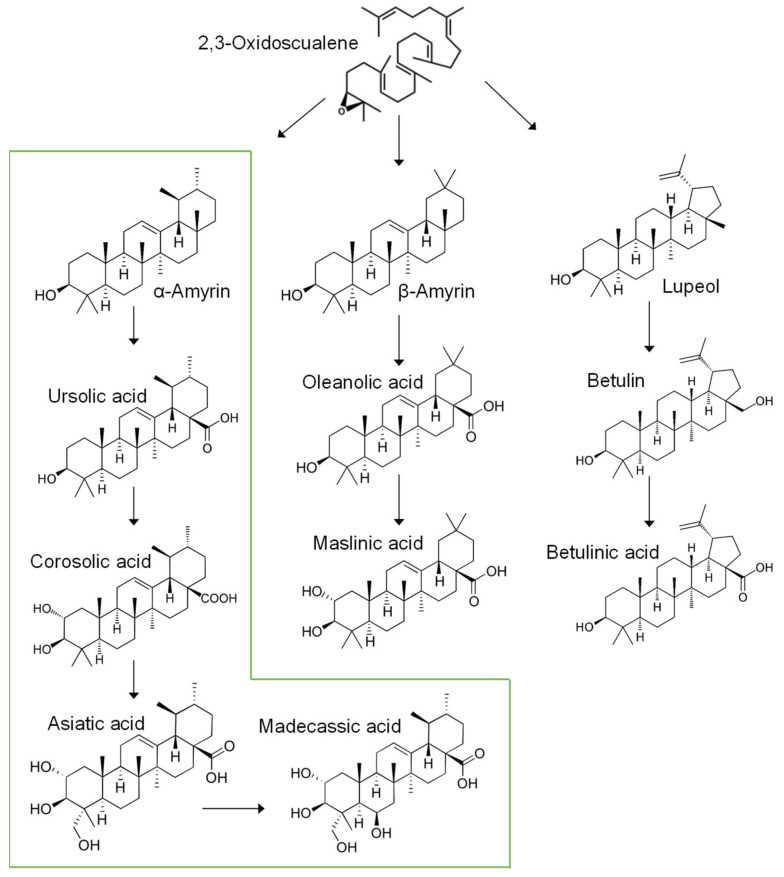
Metabolic pathways of triterpenic acids in *Syzigium formosum*. Metabolites which were detected in this study were included. The major metabolic pathway that has α-amyrin as a precursor is boxed in green.

In the anabolism of TAs, 2,3-oxidoscualene needs to be catalyzed into precursors of TAs such as amyrins (α- and β-amyrin), lupeol, dammaranediol, lanosterol, or cycloartenol. The seven TAs found in the *S. formosum* trees are categorized into three groups according to their metabolic pathway in plants (Figure 2). Ursolic acid, corosolic acid, Asiatic acid, and madecassic acid are synthesized from α-amyrin as a common precursor. β-amyrin derivatives include oleanolic acid and maslinic acid. Betulinic acid was the only TA which has lupeol as a precursor [14]. No derivatives from dammaranediol, lanosterol, or cycloartenol were detected from the *S. formosum* leaf extracts. It was notable that α-Amyrin derivatives were major TAs in all the 12 trees.

#### 2.1.2. Comparisons of Triterpenic Acids with *Centella asiatica*

**Figure 3:**
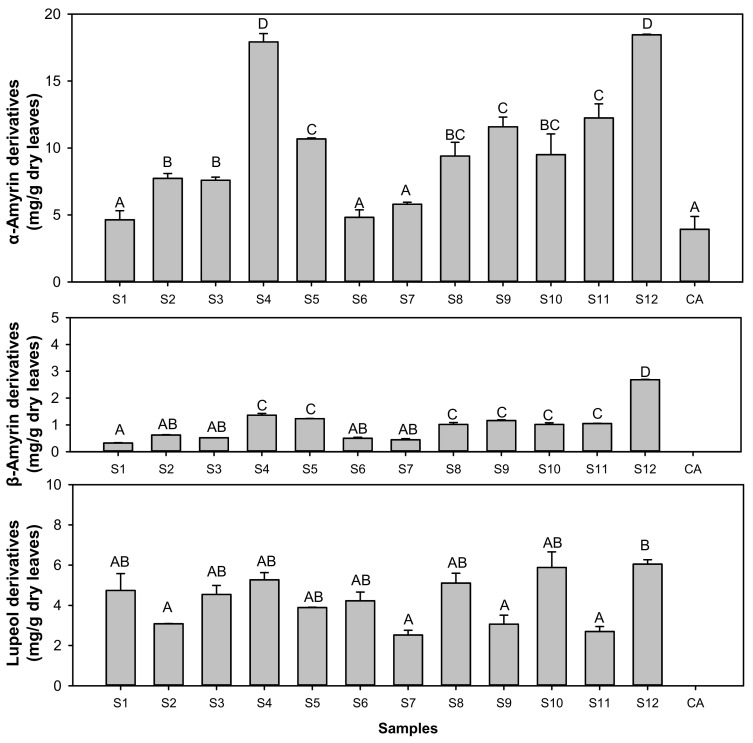
Comparison of TA amount grouped by metabolic pathways in the plant. S1-S12, *Syzygium formosum* samples; CA1 and 2, *Centella asiatica* samples. Note, no metabolite from β-amyrin and lupeol was detected in *C. asiatica*. Bars denoted with different alphabet are statistically different with *p*-value < 0.05.

Twelve trees of *S. formosum* accumulated at least seven different TAs, while *C. asiatica* contained only two Tas, asiatic acid and madecassic acid. α-Amyrin derivatives were more than 50% of the total Tas, but the β-amyrin pathway was very minor (Figure 3). Intriguingly, *S. formosum* leaves synthesized significant amount of betulinic acid with betulin which, was less than 5% of betulinic acid (data not shown).

As shown in Table 1, *S. formosum* accumulated madecassic acid, but it was only 5–10% of asiatic acid. In contrast, madecassic acid amount in the *C. asiatica* reached more than 50% of asiatic acid.

### 2.2. Antioxidative Activities and Total Phenolics

**Figure 4:**
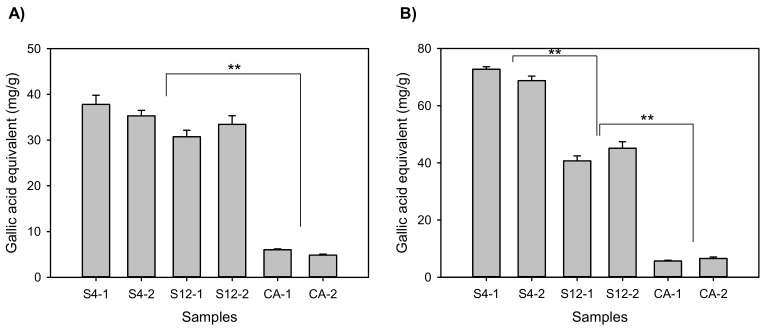
Antioxidative power (**A**) and total phenolics (**B**) of the plant extracts are expressed with gallic acid equivalent amount. S4 and S12 were the selected elite trees which accumulated the greatest amount of triterpenic acids of *Syzygium formosum* samples, and CA indicates the *Centella asiatica*. Double asterisks (**) indicate statistical difference (*p* < 0.01). Two independently prepared extracts were numbered in addition to the sample code. The assays using each extract were conducted in triplicate.

Based on the triterpenic acid amount, S4 and S12 trees were selected for further chemical and biological examinations. Before cell-based assays, antioxidative power and total phenolic compounds of S4 and S12 were determined (Figure 4). The *S. formosum* samples had about 4-fold higher antioxidant levels than the *C. asiatica*. S4 and S12 exhibited a similar level of antioxidative compounds, but S4 had a significantly larger amount of the total phenolics than S12. It is notable that the total phenolics of S12 was more than 6-fold higher than that of the *C. asiatica* sample.

### 2.3. Anti-Inflammatory Effects

#### 2.3.1. mRNA Expression of Pro-Inflammatory Genes

**Figure 5:**
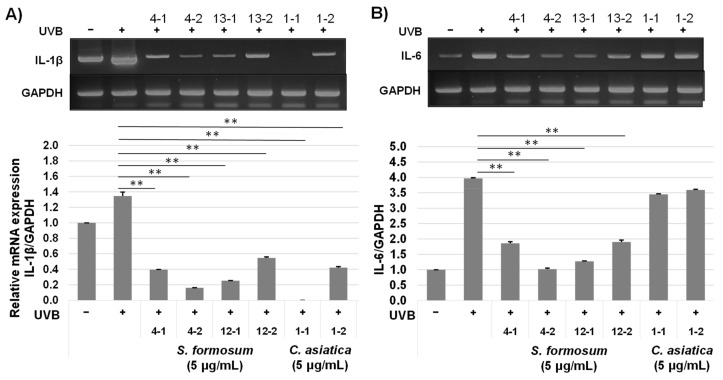

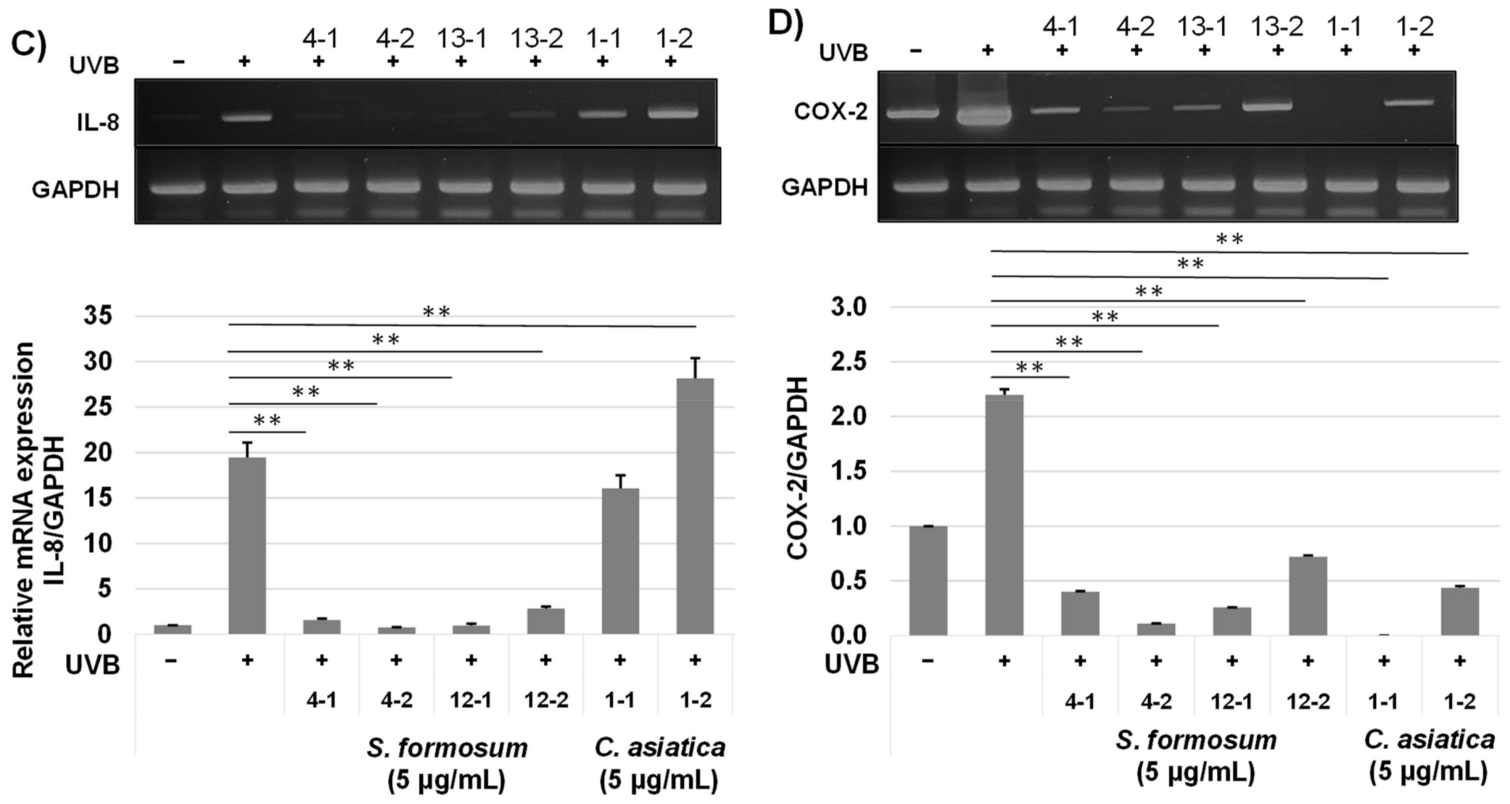
Repression of pro-inflammatory genes by *Syzygium formosum* extracts was estimated by mRNA expression. The extracts of S4 and S12 were selected based on the total triterpenic acid and compared with the *Centella asiatica* extract. The cells were exposed to 20 mJ/cm^2^ of UVB. IL-1β (**A**), IL-6 (**B**), IL-8 (**C**), and COX-2 (**D**) expressions were compared in relative amounts to a house keeping gene, glyceraldehyde 3-phosphate dehydrogenase (GAPDH). Double asterisks (**) indicate statistical difference (*p* < 0.01). Two independently prepared extracts were numbered in addition to the sample code. The assays using each extract were conducted in triplicate.

Anti-inflammatory effects of the *S. formosum* were assessed using UVB induced inflammation model of HaCaT. Based on the cytotoxicity evaluations with MTT assay (data not shown), 5 μg/mL concentration was selected for the biological activity analyses. UVB radiation greatly enhanced pro-inflammatory responses such as IL-6, IL-8, and cyclooxygenase-2 of HaCaT, but IL-1β level was not much increased. Both the *S. formosum* and the *C. asiatica* samples significantly repressed IL-1β and COX-2 expressions in the HaCaT cells that were exposed to UVB to induce inflammatory responses (Figure 5A,D). The mRNA levels of IL-6 and 8 were greatly reduced in S4 and S12 samples, while the extracts from *C. asiatica* did not produce significant changes (Figure 5B,C).

The extracts from the same sample showed slight variation, but the difference was not so great to affect the interpretation of the results. The results indicate that *S. formosum* has great anti-inflammatory effects on the UVB radiated skin cells than *C. asiatica*.

#### 2.3.2. Protein Levels of Pro-Inflammatory Cytokines

**Figure 6:**
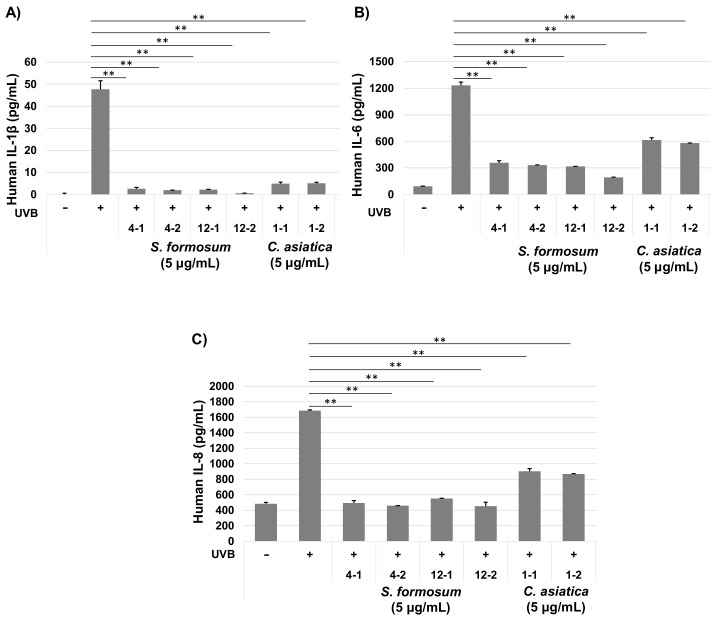
Protein levels of pro-inflammatory cytokines, IL-1β (**A**), IL-6 (**B**), and IL-8 (**C**) after a *Syzigium formosum* extract treatment. The extracts of S4 and S12 were selected based on the total triterpenic acid and compared with the *Centella asiatica* extract. The cells were exposed to 20 mJ/cm^2^ of UVB. Double asterisks (**) indicate statistical difference (*p* < 0.01). Two independently prepared extracts were numbered in addition to the sample code. The assays using each extract were conducted in triplicate.

To estimate anti-inflammatory effects of *S. formosum*, cytokine concentrations of IL-1β, IL-6 and IL-8 in HaCaT cell culture medium were analyzed. Although the mRNA level of IL-1β was not greatly increased (Figure 5A), all the cytokines in protein level were increased profoundly by UVB radiation. The extracts from S4 and S12 greatly reduced concentrations of IL-1β, IL-6, and IL-8 in the culture medium (Figure 6). Intriguingly, the *C. asiatica* sample also showed repressions of all the three cytokines. However, the anti-inflammatory effects of the *S. formosum* were significantly higher than that of the *C. asiatica*.

## 3. Discussion

In this study, 12 wild trees of *S. formosum* were analyzed to provide valuable information for industrial uses of the tree. This subtropical tree has very large and relatively thick leaves that contains large amounts of secondary metabolites. Among various phytochemicals, TAs were considered as major products based on the previous research on the chemical composition of *S. formosum* leaves, and many reports on the biological activities of TAs are available [7,9,15,16]. It is well known that secondary metabolites are affected by environmental factors as well as genetic backgrounds. Nutrients, sunlight, and temperature are the major environmental factors which manipulate the metabolism of phytochemicals [14]. In this study, wild trees in the Hanoi area were selected. Therefore, variations of temperature and sunlight might be minor. The nutrients, however, possibly affected the metabolism of *S. formosum*. In further study, we will evaluate the effects of environmental factors on the metabolism of secondary metabolites in *S. formosum*.

Initially, we found *S. formosum* leaves accumulated various TAs that are derivatives of amyrins and lupeol. As expected, lanosterol and its derivatives that are common precursors of sterol synthesis in animals and fungi were not detected in the extracts of *S. formosum*. Similar to *C. asiatica*, the most abundant TA was asiatic acid in all the 12 trees. The Asiatic acid slightly exceeded 50% of the total TAs in S4. The asiatic acid amount in dried leaves was significantly greater in the *S. formosum* samples than the *C. asiatica*. Madecassic acid, which is synthesized from asiatic acid in plants, was detected and quantified in this study, but previous studies reported no detection [10]. It is notable that glycosylated Tas were hardly detected in the *S. formosum* leaf. Harvesting conditions, such as different seasons, may affect the aglycon and glycoside composition, but this question should be answered by further studies. Among the 12 trees, S4 and S12 accumulated much greater amounts of TAs than others. Moreover, total TAs in S4 and S12 were at least 5-fold higher than the *C. asiatica*. Taken together, S4 and S12 were determined as elite trees which could be used for breeding of *S. formosum* in the near future.

To our knowledge, a genetic study of *S. formosum* has not been conducted yet. But our results indicate that triterpene anabolism is very strong in *S. formosum* leaves and favors an α-amyrin pathway. β-Amyrin, however, exhibited a very low proportion in the TA composition. Corosolic acid, which is an intermediate α-amyrin pathway to asiatic acid and betulinic acid, was relatively high in all the 12 trees. Therefore, similar to a previous report, asiatic acid, betulinic acid, and corosolic acid could be considered as standard components in the extract of *S. formosum* leaf. When combined with genetic studies, we can develop a proper strategy to control and enhance production of these secondary metabolites in *S. formosum*.

Many triterpenic acids are proved as strong bioactive compounds and used as pharmaceutical and cosmeceutical ingredients. Asiatic acid, madecassic acid, corosolic acid, and betulinic acids are representative chemicals that are well investigated and industrialized in those ways [16,17,18,19]. In this study, HaCaT cells radiated with UVB were adopted due to the well-established references of inflammatory responses of HaCaT to UVB [12,13]. Although HaCaT has dysfunctional p53, the cell shows similar inflammation responses to primary human keratinocyte. UVB radiation induces various stress responses including pro-inflammatory cytokine expressions. In a previous report, among pro-inflammatory cytokines, tumor necrosis factor-α (TNF-α) did not respond strongly when HaCaT was exposed to weak UVB [13]. This phenomenon was also found in our study (data not shown), and basal levels of pro-inflammatory cytokines in HaCaT were relatively high.

The results shown in Figure 5 and Figure 6 clearly indicate that extract of *S. formosum* leaf can repress or stop the inflammation cascade in skin induced by UVB radiation. Low concentration of *S. formosum* leaf extracts reduced IL-1β, IL-6, and IL-8 at a similar level to the control group, which showed basal level of cytokines. IL-1β is one of the major cytokines produced by stratum corneum and fibroblast [20,21]. It activates an inflammatory response of epithelial cells and induces differentiation of Th17. IL-6 has a major role in the acute inflammatory response of the liver. In human skin, a high level of IL-6 induces fibrosis by complex interplay with TFG-β in dermal cells [22]. IL-8 is produced mainly by macrophage, epithelial cells, and airway smooth muscle cells. This cytokine is a neutrophil chemotactic factor and stimulates phagocytic functions of that immune cell. In a recent study, IL-1β and IL-8 were found to be highly expressed cytokines in the stratum corneum of atopic dermatitis lesional skin [21]. In addition, the level of IL-1β showed a positive correlation with severity of the disease. Increased expression of COX-2 can induce premature aging of a transgenic mouse model [23].

The *S. formosum* samples contained various components as well as seven TAs [10], therefore, anti-inflammatory functions of the extract were derived from the complex of the phytochemicals. However, it is plausible that the TAs are the major functional components due to their high concentration in the extract. The total TAs in the extract were around 15% (S4) to 25% (S12) of total dry matter content of the extract. The major TA in *S. formosum*, asiatic acid, is well recognized by its strong anti-oxidative, anti-inflammatory, and anti-allergic effects. Corosolic acid inhibits inflammation of adipose tissue in a high-fat diet mouse model. Recently, protecting effects of corosolic acid on the diabetic renal damage were reported [17]. One of the major TAs in the *S. formosum* leaf, betulinic acid, also has potent anti-inflammatory and anti-cancer effects. In a lethal endodoxemia mouse model, feeding of betulinic acid significantly decreased TNF-α and increased IL-10 [24]. Recently, anti-cancer functions of betulinic acid on the glioblastoma were reported [18]. Regarding contents of TAs, *S. formosum* leaf is a good raw material to provide the biological benefits of asiatic acid, corosolic acid, and betulinic acid to human health.

Taken all together, we have shown that *S. formosum* leaves contained greater compositions of biologically active phytochemicals. The extract of *S. formosum* leaves showed stronger anti-inflammatory effects than *C. asiatica* on human keratinocyte, HaCaT. In further studies, biological activities on the skin of *S. formosum* leaves will be conducted to focus on anti-wrinkle activities and improvements of skin barrier functions.

## 4. Materials and Methods

### 4.1. Plant Samples and Reagents

Leaves of *S. formosum* were harvested and sun-dried in September–October, 2020. Wild trees older than 5 years were selected in the Hanoi area, Vietnam. Dried *C. asiatica* from Indonesia was purchased online (Jung Woo-dang, Seoul, Korea). Cell culture reagent, Dulbecco Modified Eagle Medium (DMEM), was purchased from WELGENE (Daegu, Korea). Penicillin, streptomycin, and 0.05% trypsin-EDTA were purchased from Gibco (Waltham, MA, USA). Reverse transcription mix (5x master premix, Elpis-Biotech, Daejeon, Korea) and Taq PCR premix (AccuPower ProFi, Bioneer, Daejeon, Korea) were used for gene works. HPLC analysis grade asiatic acid, madecassic acid, corosolic acid, betulinic acid, oleanolic acid (4 chemicals: Sigma-Aldrich, St. Louis, MO, USA), ursolic acid (Tokyo Chemical Industry, Tokyo, Japan), and maslinic acid (Chengdu Biopurify Phytochemicals Ltd., Chengdu, China) with at least 95% purity were used.

### 4.2. Extraction of Plants

Dried leaves of *S. formosum* or *C. asiatica* containing 6–11% water were ground using a home blender. Then, 2 g of each sample was mixed with 40 mL of 70% ethanol at 50 °C for 24 h. The supernatant was separated and harvested from insoluble matters by centrifugation at 4 °C (12,000× *g*) for 20 min. The extracts were freeze-dried to powder and kept at −18 °C before use.

### 4.3. Instrumental Analyses of Triterpenic Acids

Sample pretreatment for liquid chromatography-mass spectrometry (LC-MS) was conducted using solid-phase extraction (C18 cartridge, 200 mg, 3 mL, Waters, Milford, MA, USA) to remove hydrophilic components, such as sugars. Recovery of this was 90–105%. UPLC system (Agilent 1290 Infinity, Agilent Technologies Inc., Santa Clara, CA, USA) with a triple quadrupole mass spectrometer (Agilent 6470, Agilent Technologies Inc.), equipped with a turbo ion spray source was used for the identification and quantification of TAs. C18 column (2.1 × 100 mm, 1.8 µm, Zorbax Eclipse Rapid Resolution High Definition, Agilent Technologies Inc.) was stabilized with 5 mM ammonium formate (solvent A), and analytes were eluted with a mixture of 95% methanol and 5% isopropyl alcohol with 5 mM ammonium formate (solvent B). The flow rate was 0.2 mL/min with gradient of solvent B: 0–1 min, 70%; 1–3 min, 80%; 3–8 min, 82%; 8–20 min, 84%; and 20–25.5 min, 100%. The gas flow and temperature were 10 L/min and 270 °C, respectively. The nebulizer was set as 40 psi, and the sheath gas temperatures was 300 °C. Electrospray ionization with voltage of 3500 V was used with the positive mode. Multiple reaction monitoring (MRM) was adapted for quantification of TAs. Seven standard molecules were analyzed by the same method including solid-phase extraction.

### 4.4. Chemical and Biological Activity Assay

#### 4.4.1. Antioxidative Activity

Reducing power of the plant extracts was determined by DPPH method. The assay was conducted as described previously [25]. Briefly, 0.95 mL of 0.2 mM DPPH solution was mixed with 0.05 mL of the plant extract which was diluted appropriately and incubated in the dark and at room temperature for 30 min. Absorbance of the reacted sample was measured at 517 nm. In this study, gallic acid was used as a standard molecule.

#### 4.4.2. Total Phenolic Compounds

Total phenolic compounds were determined by the Folin–Ciocalteu reaction method. Overall assay was conducted as described previously with slight modification [26]. First, the plant extracts were properly diluted. The diluted sample 0.1 mL, distilled water 1.5 mL, and the Folin–Ciocalteu solution were mixed and incubated for 5 min. After adding 20% sodium carbonate 0.3 mL, the mixture stood at room temperature for 30 min. Then, absorbance at 765 nm was measured. Gallic acid was used as a standard material.

#### 4.4.3. Cell Culture and Viability Assay

The HaCaT cell line was kindly provided by Dr. J.M. Shin at Research Institute for Medical Science, College of Medicine, Chungnam National University, Korea. Cytotoxicity of the plant extract was evaluated with 3-(4,5-dimethylthiazol-2-yl)-2,5-diphenyltetrazolium bromide (MTT; Sigma-Aldrich Co., St Louis, Mo, USA) assay. Briefly, the cells (0.2 × 10^5^/mL) were seeded on a 96-well plate and cultured overnight. The plant extract was first dissolved in DMSO and adjusted with the culture medium to the final concentration. The properly diluted DMSO with the culture medium, as a vehicle, was mixed into the culture plate. After 24 h incubation, the MTT solution was added and incubated for a further 3 h. The supernatant was removed, then 0.1 mL of DMSO was added. Finally, the absorbance at 540 nm was measured with microplate reader (Epoch, Biotek, Winooski, VT, USA).

#### 4.4.4. Anti-Inflammatory Effects of Plant Extracts

Firstly, the UVB radiation condition was optimized to induce pro-inflammatory cytokines (IL-1β, IL-6, and IL-8) and COX-2. A range of UVB radiation, 5–40 mJ/cm^2^, was tested and 20 mJ/cm^2^ was selected for further experiment condition.

HaCaT cells (1.0 × 10^6^/mL) were seeded in a 6-well plate and incubated overnight. Six hours before UVB exposure, the plant extract was treated on the cells at the final concentration of 5 μg/mL. After removing medium, 2 mL serum free-media (DMEM), having the same concentration of the plant extract, was added to the cells. The cells were incubated for 18 h, then harvested for total RNA extraction. The cDNA was prepared from the total RNA and used for the PCR to determine mRNA expression of IL-1β, IL-6, IL-8, and COX-2. The expression levels of the target genes were compared with a house keeping gene, actin. Sequences of PCR primers are shown in Table 2.

For the ELISA, the supernatants were collected, and the concentrations of cytokines correspondingly quantified. ELISA assays for IL-1β, IL-6, and IL-8 were executed with a Thermo Fisher Scientific ELISA system (Thermo Fisher Scientific, Inc., Waltham, MA, USA) according to the protocols from the manufacturer.

### 4.5. Statistical Analyses

Statistical analyses between the control group and the plant extract treatment group were conducted using one-way ANOVA. The statistical significance was indicated by * for *p* < 0.05 and ** for *p* < 0.01, respectively.

## Figures and Tables

**Table 1:** Triterpenic acids in twelve *Syzygium formosum* trees. Some minor components that were close to the limits of quantification were excluded. For the comparison, two samples of *Centella asiatica* were analyzed by the same method.

*S. formosum*	Triterpenic Acids ^a^ (mg/g Dry Weight)							Total
	Madecassic Acid	Asiatic Acid	Maslinic Acid	Corosolic Acid	Betulinic Acid	Oleanolic Acid	Ursolic Acid	
S1	0.20 ± 0.02	3.36 ± 0.51	0.26 ± 0.01	0.89 ± 0.15	4.74 ± 0.84	0.065 ± 0.005	0.18 ± 0.01	9.695 ± 1.545
S2	0.67 ± 0.01	5.26 ± 0.25	0.53 ± 0.02	1.59 ± 0.12	3.09 ± 0.02	0.10 ± 0.01	0.23 ± 0.01	11.44 ± 0.39
S3	0.50 ± 0.01	5.57 ± 0.17	0.41 ± 0.01	1.25 ± 0.06	4.54 ± 0.45	0.12 ± 0.01	0.28 ± 0.01	12.65 ± 0.68
S4	0.98 ± 0.04	12.6 ± 0.5	1.04 ± 0.05	3.54 ± 0.04	5.27 ± 0.36	0.33 ± 0.2	0.81 ± 0.04	24.56 ± 1.05
S5	0.37 ± 0.02	5.89 ± 0.02	0.98 ± 0.01	3.76 ± 0.12	3.89 ± 0.02	0.26 ± 0.01	0.66 ± 0.01	15.81 ± 0.18
S6	0.25 ± 0.04	2.78 ± 0.29	0.44 ± 0.04	1.64 ± 0.22	4.23 ± 0.43	0.065 ± 0.005	0.16 ± 0.01	9.56 ± 1.03
S7	0.24 ± 0.02	3.99 ± 0.05	0.37 ± 0.04	1.35 ± 0.07	2.53 ± 0.24	0.08 ± 0.01	0.23 ± 0.02	8.77 ± 0.43
S8	0.44 ± 0.03	4.68 ± 0.54	0.84 ± 0.06	3.78 ± 0.42	5.11 ± 0.50	0.18 ± 0.01	0.52 ± 0.05	15.53 ± 1.59
S9	0.29 ± 0.01	6.81 ± 0.46	0.95 ± 0.03	3.83 ± 0.30	3.07 ± 0.45	0.22 ± 0.02	0.66 ± 0.03	15.81 ± 1.18
S10	0.39 ± 0.03	4.51 ± 0.80	0.84 ± 0.05	4.02 ± 0.70	5.89 ± 0.78	0.19 ± 0.02	0.60 ± 0.03	16.41 ± 2.38
S11	0.27 ± 0.02	7.76 ± 0.61	0.86 ± 0.01	3.66 ± 0.47	2.70 ± 0.25	0.19 ± 0.02	0.56 ± 0.01	15.99 ± 1.35
S12	0.82 ± 0.01	10.07 ± 0.09	2.16 ± 0.02	6.21 ± 0.01	6.05 ± 0.22	0.53 ± 0.03	1.36 ± 0.02	27.19 ± 0.26
Average	0.42 ± 0.25	5.84 ± 2.81	0.79 ± 0.47	2.95 ± 1.50	4.24 ± 1.20	0.19 ± 0.12	0.51 ± 0.32	14.94 ± 5.50
*C. asiatica*	1.72 ± 0.26	3.21 ± 0.7	NA ^b^	NA	NA	NA	NA	4.94 ± 0.96

^a^ The order of chemicals follows retention times of triterpenic acids in HPLC-MS/MS analyses. ^b^ Not available: the chemical was not detected or under the limits of quantification.

**Table 2:** PCR primers of pro-inflammatory genes and actin.

Gene	Forward	Reverse
IL-1β	GGG CCT CAA GGA AAA GAA TC	TTC TGC TTG AGA GGT GCT GA
IL-6	AGG AGA CTT GCC TGG TGA AA	CAG GGG TGG TTA TTG CAT CT
IL-8	TCT GTG TGA AGG TGC ACT T	AGC CCT CTT CAA AAA CTT CT
COX-2	GGT CTG GTG CCT GGT CTG ATG	GTC CTT TCA AGG AGA ATG GTG C
Actin	GGC GGA CTA TGA CTT AGT TG	AAC AAC AAT GTG CAA TCA A

## Data Availability

All the data supporting this article were included in the main text.
